# Echocardiographic diagnosis, management and monitoring of pulmonary embolism with right heart thrombus in a patient with myotonic dystrophy: a case report

**DOI:** 10.1186/1476-7120-8-18

**Published:** 2010-05-16

**Authors:** Bernd Hewing, Leyli Ghaeni, Henryk Dreger, Eva M Fallenberg, Alexander Panda, Gert Baumann, Adrian C Borges

**Affiliations:** 1 Medizinische Klinik für Kardiologie und Angiologie, Campus Mitte, Charité - Universitätsmedizin Berlin, Germany; 2 Klinik für Neurologie, Campus Mitte, Charité - Universitätsmedizin Berlin, Germany; 3 Institut für Radiologie, Campus Mitte, Charité - Universitätsmedizin Berlin, Germany; 4 Department of Internal Medicine, Yale University School of Medicine, New Haven, CT, USA

## Abstract

Acute pulmonary embolism (PE) is a common disease which frequently results in life-threatening right ventricular (RV) failure. High-risk PE, presenting with hypotension, shock, RV dysfunction or right heart thrombus is associated with a high mortality, particularly during the first few hours. Accordingly, it is important to commence effective therapy as soon as possible. In the case described in this report, a 49-year-old woman with myotonic dystrophy type 1 presented with acute respiratory failure and hypotension. Transthoracic echocardiography showed signs of right heart failure and a mobile right heart mass highly suspicious of a thrombus. Based on echocardiographic findings, acute thrombolysis was performed resulting in hemodynamic stabilization of the patient and complete resolution of the right heart thrombus. This case underscores the important role of transthoracic echocardiography for the diagnosis, management and monitoring of PE and underlines the efficacy and safety of thrombolysis in the treatment of PE associated with right heart thrombus.

## Background

Right sided heart thrombi can be found in 4-18% of patients presenting with acute pulmonary embolism [1,2]. They may develop within the right heart chambers or origin from peripheral venous clots that got stuck in right heart structures on their way to the lungs. Type A thrombi have a worm like shape, are extremely mobile and mostly represent peripheral venous clots which temporarily lodge into the right heart. Due to their extreme mobility these clots are at high risk for severe and often fatal pulmonary embolism. Type B thrombi which are morphologically similar to the left heart thrombi attach to the right atrial or ventricular wall indicating that these thrombi mostly develop within the right heart [3,4]. The presence of a right heart thrombus in PE is relevant for the prognosis as it predicts a higher mortality rate (> 44%) [5].

Here, we present a case where transthoracic echocardiography was used for the diagnosis, management and follow-up of pulmonary embolism with right heart thrombus.

## Case presentation

**Figure 1:**
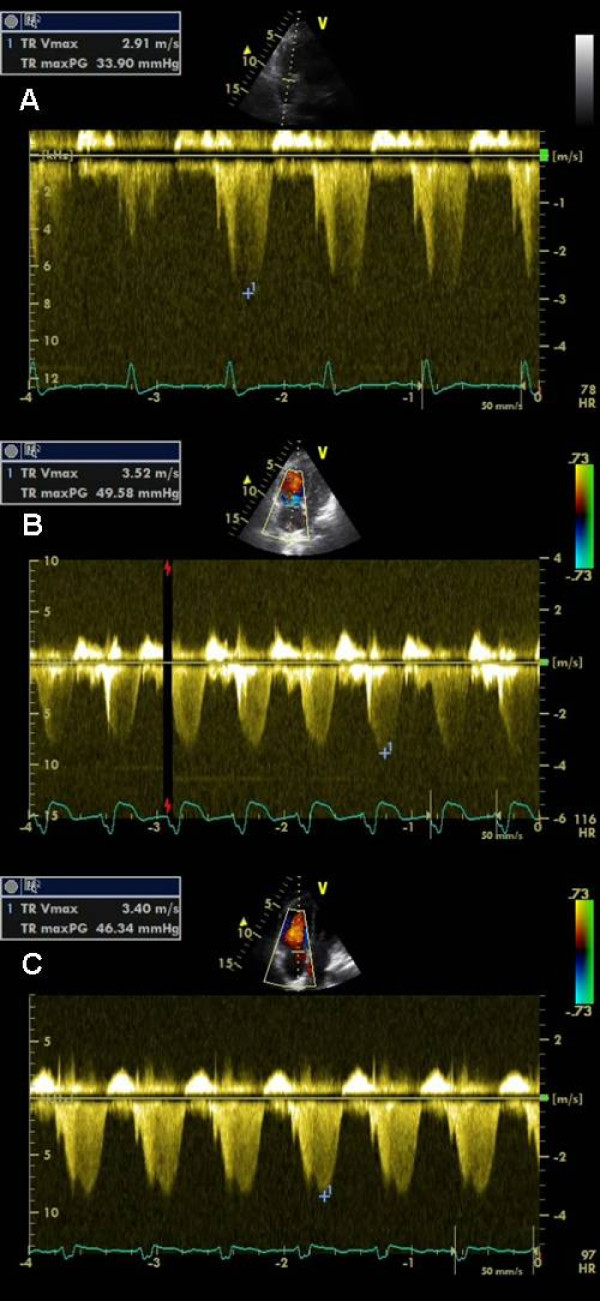
**Pulmonary artery pressure (PAP)**. Continuous-wave Doppler echocardiography was used to estimate PAP: **(A) **on admission, **(B) **during the acute phase of pulmonary embolism with right heart thrombus, and **(C) **on day 2 after thrombolysis.

A 49-year-old woman with myotonic dystrophy type 1 (MD1, Curschmann-Steinert Syndrom) was admitted to our neurological intensive care unit because of respiratory failure due to myoneuronal hypoventilation and clinical suspicion of pneumonia. Due to progressive respiratory decompensation she had to be intubated prior to admission. At arrival, her vital signs were as follows: body temperature 37°C, blood pressure 130/70 mmHg, pulse 70 beats per minute (bpm) and respiratory rate 14 breaths per minute. Laboratory tests showed moderately elevated CRP (10.95 mg/dl; normal < 0.5 mg/dl) and normal white blood cell counts. Blood and urine cultures drawn at this time were ultimately found to be negative. D-dimer was elevated (4.81 mg/l; normal < 0.5 mg/l). Computed tomography (CT) of the chest was performed showing focal consolidations and ground glass opacity in the left lower lobe as signs of an evolving pneumonia. There was a filling defect in the right upper lobe pulmonary artery highly suggestive of a pulmonary embolus (See Additional file 1). Echocardiography showed a normal systolic and diastolic function of the left ventricle. There was mild regurgitation of the mitral and pulmonary valve and moderate regurgitation of the tricuspid valve. Systolic pulmonary artery pressure (PAP) estimated by measurement of systolic tricuspid regurgitation velocity was 34 mmHg plus central venous pressure (CVP) (Figure 1A) (See Additional file 2). Therapeutic anticoagulation with low-molecular-weight heparin was initiated and an antibiotic therapy with ceftriaxon and azithromycin was continued.

On the sixth day, the respiratory and hemodynamic situation of the patient worsened acutely. The monitor ECG showed sinus tachycardia with 130 bpm, blood pressure was 50/30 mmHg, and the oxygen saturation decreased to 60%. The patient could only be stabilized by adjustment of ventilation parameters (controlled ventilation: bilevel positive airway pressure mode) and high doses of vasopressors.

**Figure 2:**
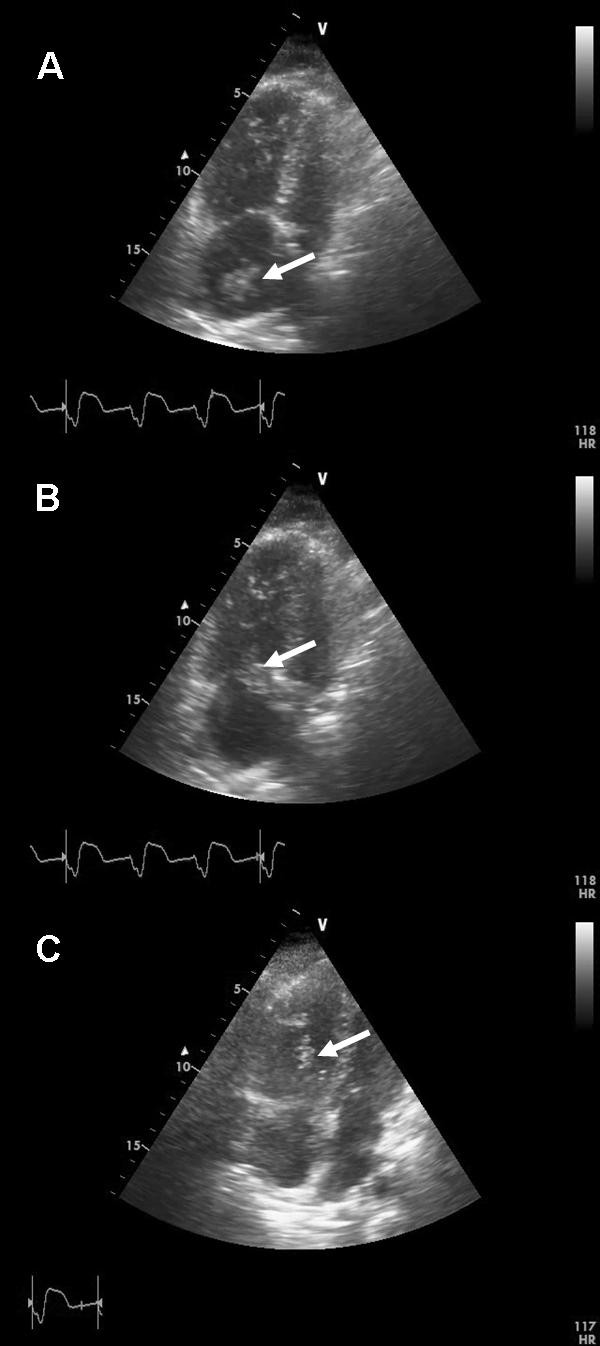
**Transthoracic echocardiogram (TTE) with a right sided heart thrombus**. Apical 4-chamber view with **(A) **a mobile thrombus (marked by arrow) in the right atrium moving through **(B) **the tricuspid orifice into **(C) **the right ventricle.

Bedside transthoracic echocardiography was performed immediately, showing a mobile mass (3 cm × 2 cm), highly suspicious of a thrombus, moving back and forth from the right atrium to the ventricle through the tricuspid orifice during cardiac cycle. The right ventricle was severely dilated combined with right ventricular mid free-wall hypo-/akinesis and nearly normal contraction of the apical segment (McConnell sign) [6]. Tricuspid annular plane systolic excursion (TAPSE) was profoundly impaired (9 mm). Tricuspid regurgitation was severe and systolic PAP was markedly elevated (50 mmHg plus CVP) (Figure 1B and 2) (See Additional file 3). Based on the echocardiographic findings and on the hemodynamic instability of the patient, therapeutic decision for acute thrombolysis was made. A bolus of 20 mg recombinant tissue plasminogen activator (rt-PA) was given intravenously followed by 100 mg over two hours. Intravenous fluid was administered rapidly. The hemodynamic situation of the patient stabilized over the following hour.

**Figure 3:**
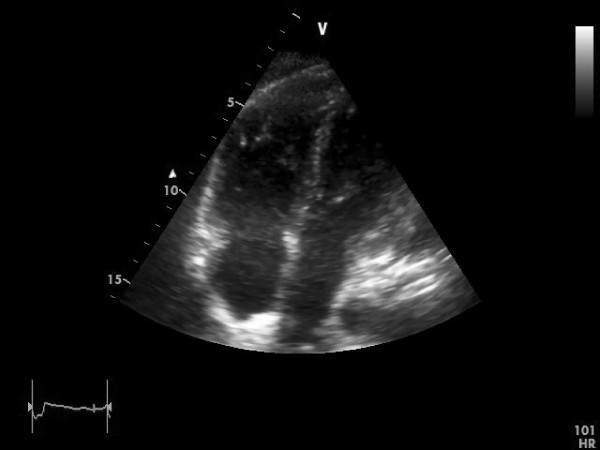
**TTE on day 2 after thrombolytic therapy**. Apical 4-chamber view shows complete disappearance of the right heart thrombus.

Echocardiography on day two after thrombolytic therapy showed a complete resolution of the right sided heart thrombus (Figure 3) See Additional file 4. Right ventricular mid free wall kinetic and function (TAPSE 16 mm) have improved markedly. There was only a slight improvement of systolic PAP seen on echocardiogram on day two (46 mm Hg plus CVP) and day 14 (44 mm Hg plus CVP) after thrombolytic therapy (Figure 1C). CT scan of the chest two days after thrombolytic therapy showed no severe pulmonary embolism indicating that thrombolytic therapy was successful and the disappearance of the right heart thrombus was rather due to *in situ *lysis than due to migration to the pulmonary arteries. Duplex ultrasonography of extremity veins revealed deep vein thrombosis of the left superficial femoral vein, profunda femoris vein and popliteal vein.

Heparin-induced thrombocytopenia type II and activated protein C-resistance could be excluded.

## Discussion

Myotonic Dystrophy Type 1 is an autosomal dominant multisystem disorder caused by a mutated expansion of a CTG repeat in the 3' untranslated region of a serine-threonine kinase gene on chromosome 19 [7]. MD1 patients present with myotonia, distal weakness, frontal baldness, diabetes, megacolon, infertility, and polychromatic cataract. However, the most disabling feature of MD1 is muscle wasting that leads to immobilization and hypoventilation [8]. The most frequent cardiac manifestations include myocardial thickening, heart failure, AV-block, and *torsades de pointes*-tachycardia [9]. Because of the inevitable immobilization of patients with myotonic dystrophy they are believed to have a higher risk of thrombosis. Despite this fact only few cases have been published on thrombosis associated with myotonic/muscular dystrophy. A small study of 69 patients with Duchenne muscular dystrophy in Japan found abnormal coagulation tests in 96% of the patients. The authors suggest that unknown inherent factors, immobilization and cardiac dysfunction have an important impact on the development of a hypercoagulable state in this disease [10].

Acute pulmonary embolism (PE) is a common disease that may lead to life-threatening right ventricular failure. Computed tomography of the chest is the main diagnostic approach for the detection of PE. However, in a highly unstable patient as in our case - where transportation of the patient is not feasible - diagnosis of PE may be accepted solitary on the basis of direct or indirect echocardiographic findings. Additionally, duplex ultrasonography of the extremities - especially in the presence of central venous catheters - might be helpful to confirm the diagnosis.

High-risk PE, defined by hypotension, shock or RV dysfunction, is associated with a high mortality, particularly during the first few hours. Accordingly, it is important to commence effective therapy as soon as possible. In stable patients, therapy with unfractionated or low-molecular-weight heparin is recommended. According to current ESC guidelines, thrombolytic therapy is the first-line treatment (unless there are contraindications for thrombolysis) in addition to heparin for patients with high-risk PE presenting with cardiogenic shock and/or persistent arterial hypotension [11].

While therapeutic strategies for the treatment of high-risk PE are well defined, the optimal therapy for a right heart thrombus in PE - thrombolysis, heparin alone or embolectomy - remains an open and controversial debate. A meta-analysis published in 2002 points to an advantage of thrombolytic therapy in reducing mortality compared to anticoagulation with heparin or surgical embolectomy [12]. A more recent prospective study found a good outcome and rapid improvement of echocardiographic and scintigraphic parameters after thrombolysis with rt-PA in 9 patients with mobile right heart thrombus and massive PE [13]. In the ICOPER registry, thrombolytic treatment was the preferred option but the 14-day mortality was still high (20.8%) (Table 1) [1]. An additional advantage of thrombolytic therapy is the simultaneous thrombolysis of cardiac and pulmonary arterial thrombi as well as of deep vein thrombosis, which may be responsible for recurring of embolisms. In our case, thrombolytic therapy with rt-PA worked quickly, effectively and safely resulting in a complete dissolution of the cardiac thrombus.

**Table 1:** Effect of treatment on mortality in pulmonary embolism associated with right heart thrombus.

Author	n	PE	Follow-up	Mortality		
				Thrombolysis	Heparin	Embolectomy
Torbicki et al. 2003 [1] (ICOPER Registry)	42	Yes	14 days/3 months	20.8%/29.2%	23.5%/29.4%	25.0%/25.0%
Ferrari et al. 2005 [15]	16	Yes	30 days	0%	-	-
Rose et al 2002 [12] Meta-analysis	177	Yes (98%)	NA	11.3%	28.6%	23.8%

n = number of patients, PE = pulmonary embolism, NA = not available

Despite rapid recovery of right ventricular function after thrombolytic therapy only a slight improvement of systolic PAP was seen in our patient during the two weeks follow-up. However, this short term follow-up might not be representative for the long-term outcome regarding pulmonary arterial pressure. Ribeiro et al. reported in their echocardiography study of 78 patients with acute PE and increased PAP that a stable phase of systolic PAP is achieved within 38 days after diagnosis of acute PE. The treatment (heparin versus thrombolysis) received in the acute phase resulted in no significant difference in the time to achieve the stable phase or in the level of systolic PAP estimated for the stable phase. A systolic PAP of > 50 mmHg at the time of diagnosis of acute PE was associated with persistent pulmonary hypertension after one year [14].

## Conclusions

Our patient was diagnosed with massive PE according to clinical symptoms and echocardiography showing right heart failure and a right heart thrombus. Based on these findings, acute thrombolysis was performed resulting in hemodynamic stabilization of the patient and complete resolution of the right heart thrombus. Echocardiography was the only possible diagnostic approach as transportation of the patient for a CT scan was not feasible due to hemodynamic instability. Transthoracic echocardiography is a fast, practical and sensitive approach for the detection of right ventricular dysfunction and right heart thrombus. Therefore it should be performed as soon as possible in unstable patients with suspected PE.

The present case underscores the important role of transthoracic echocardiography for the diagnosis, management and monitoring of PE. It also underlines the efficacy and safety of thrombolysis in the treatment of PE associated with right heart thrombus.

## Consent

Written informed consent was obtained from the patient's legal representative for publication of this case report and any accompanying images. A copy of the written consent is available for review by the Editor-in-Chief of this journal.

## List of abbreviations

CVP: central venous pressure; MD1: myotonic dystrophy type 1, Curschmann-Steinert Syndrom; PAP: pulmonary artery pressure; PE: pulmonary embolism; rt-PA: recombinant tissue plasminogen activator; RV: right ventricular; TAPSE: tricuspid annular plane systolic excursion; TTE: transthoracic echocardiogram.

## Competing interests

The authors declare that they have no competing interests.

## Authors' contributions

BH conceived the case report, performed bedside echocardiographic examinations, reviewed literature and wrote the manuscript. HD performed bedside echocardiographic examinations. LG, HD and ACB have been involved in drafting the manuscript. EMF provided radiologic assistance. AP contributed critical revision of the manuscript. GB has supervised and commented the manuscript. ACB was the supervisor of echocardiographic examinations, head of the echocardiography lab, and contributed by revising the manuscript critically. All authors read and approved the final manuscript.

## Supplementary Material

Additional file 1 **Computed tomography (CT) of the chest on admission**. Contrast-enhanced CT scan of the chest showing a filling defect in the right upper lobe pulmonary artery (marked by arrow) indicative of a pulmonary embolus and bilateral pleural effusions; **(A) **transversal view, **(B) **coronal view. Click here for file

Additional file 2 **Transthoracic echocardiogram (TTE) on admission**. Apical 4-chamber view. Click here for file

Additional file 3 **TTE with a right sided heart thrombus**. Apical 4-chamber view with a mobile thrombus in the right atrium moving through the tricuspid orifice into the right ventricle. Click here for file

Additional file 4 **TTE on day 2 after thrombolytic therapy**. Apical 4-chamber view shows complete disappearance of the right heart thrombus. Click here for file
