# Supplementary figures and images for: Echocardiographic diagnosis, management and monitoring of pulmonary embolism with right heart thrombus in a patient with myotonic dystrophy: a case report
Source: Cardiovasc Ultrasound. 2010 May 16;8:18. doi: 10.1186/1476-7120-8-18 (PMC2887806; doi:10.1186/1476-7120-8-18)

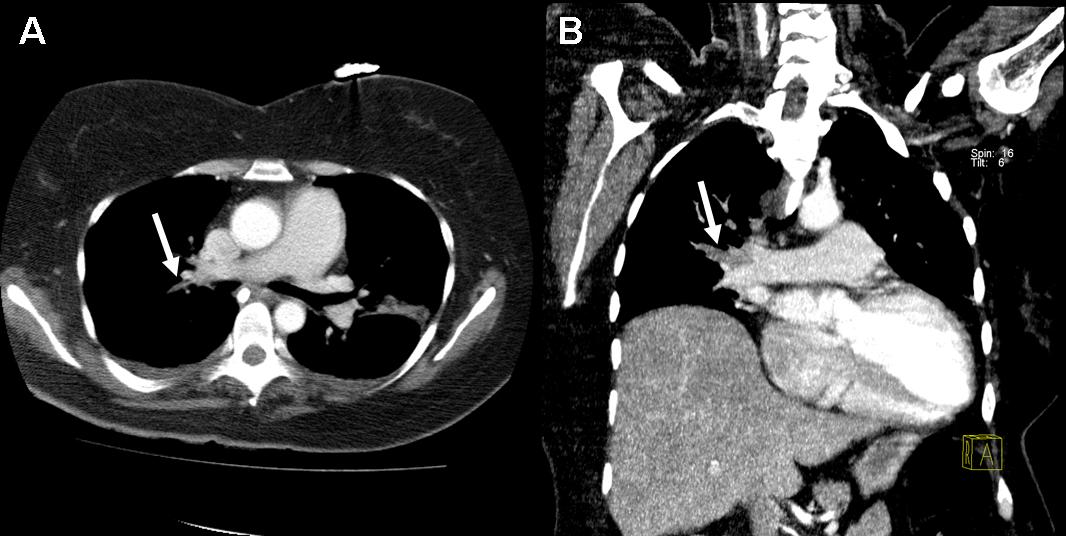

Supplement: Additional file 1 — Computed tomography (CT) of the chest on admission. Contrast-enhanced CT scan of the chest showing a filling defect in the right upper lobe pulmonary artery (marked by arrow) indicative of a pulmonary embolus and bilateral pleural effusions; (A) transversal view, (B) coronal view. [file 1476-7120-8-18-S1.JPEG]
